# Induced Fungal Resistance to Insect Grazing: Reciprocal Fitness Consequences and Fungal Gene Expression in the *Drosophila*-*Aspergillus* Model System

**DOI:** 10.1371/journal.pone.0074951

**Published:** 2013-08-30

**Authors:** Silvia Caballero Ortiz, Monika Trienens, Marko Rohlfs

**Affiliations:** 1 Johann-Friedrich-Blumenbach Institute for Zoology and Anthropology, Georg-August-University Göttingen, Göttingen, Germany; 2 Evolutionary Genetics, Centre for Ecological and Evolutionary Studies, University of Groningen, Groningen, The Netherlands; 3 Department of Animal Evolutionary Ecology, Institute for Evolution and Biodiversity, University of Münster, Münster, Germany; University of Utah, United States of America

## Abstract

**Background:**

Fungi are key dietary resources for many animals. Fungi, in consequence, have evolved sophisticated physical and chemical defences for repelling and impairing fungivores. Expression of such defences may entail costs, requiring diversion of energy and nutrients away from fungal growth and reproduction. Inducible resistance that is mounted after attack by fungivores may allow fungi to circumvent the potential costs of defence when not needed. However, no information exists on whether fungi display inducible resistance. We combined organism and fungal gene expression approaches to investigate whether fungivory induces resistance in fungi.

**Methodology/Principal Findings:**

Here we show that grazing by larval fruit flies, *Drosophila melanogaster*, induces resistance in the filamentous mould, *Aspergillus nidulans*, to subsequent feeding by larvae of the same insect. Larval grazing triggered the expression of various putative fungal resistance genes, including the secondary metabolite master regulator gene *laeA*. Compared to the severe pathological effects of wild type *A. nidulans*, which led to 100% insect mortality, larval feeding on a *laeA* loss-of-function mutant resulted in normal insect development. Whereas the wild type fungus recovered from larval grazing, larvae eradicated the chemically deficient mutant. In contrast, mutualistic dietary yeast, *Saccharomyces cerevisiae*, reached higher population densities when exposed to *Drosophila* larval feeding.

**Conclusions/Significance:**

Our study presents novel evidence that insect grazing is capable of inducing resistance to further grazing in a filamentous fungus. This phenotypic shift in resistance to fungivory is accompanied by changes in the expression of genes involved in signal transduction, epigenetic regulation and secondary metabolite biosynthesis pathways. Depending on reciprocal insect-fungus fitness consequences, fungi may be selected for inducible resistance to maintain high fitness in fungivore-rich habitats. Induced fungal defence responses thus need to be included if we wish to have a complete conception of animal-fungus co-evolution, fungal gene regulation, and multitrophic interactions.

## Introduction

Fungi have diverse physiochemical and chemical properties that appear to be favoured by natural selection because they mediate resistance to fungivory, i.e. they harm or repel fungivores [1–5]. Despite increasing evidence of such sophisticated defences against fungivores, it is still unclear whether fungi primarily invest energy and resources in defensive traits regardless of the presences of fungivores (*constitutive resistance*) or whether they have evolved, in analogy to herbivore-plant interactions [6], the ability to show phenotypic variation in response to attack (*inducible resistance*). Inducible defences would endow fungi with the possibility of allocating resources in an “economy-friendly” way and may have strong influence on multitrophic interactions [7].

**Figure 1:**
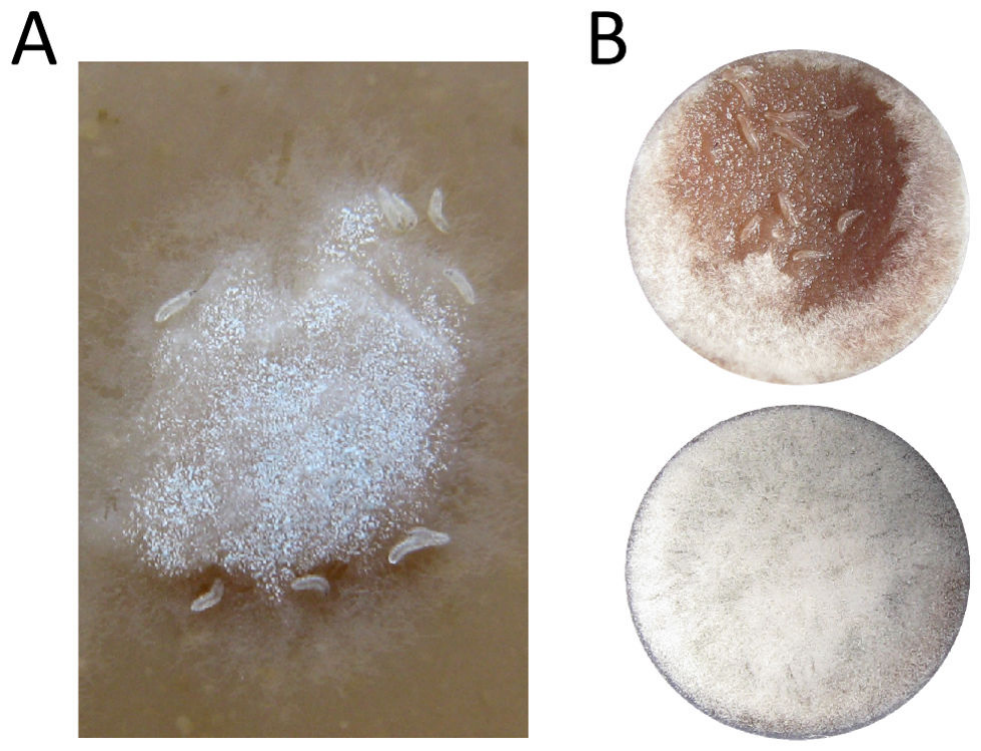
*Drosophila melanogaster* larval grazing on *Aspergillus nidulans*. (A) *D*. *melanogaster* larvae are attracted to *A*. *nidulans* colonies. Larvae are about 1 mm. (B) Eradication of *A*. *nidulans* hyphal tissue by *D*. *melanogaster* larvae (top), and fungal growth under undisturbed conditions (bottom). Images depict fungal development after 72 h incubation at 25°C on nutrient-poor fruit agar. Arena diameter: 10 mm.

We use the *Drosophila* - *Aspergillus* insect-fungus model system [8] to investigate inducible resistance to fungivory by fungi. Drosophilid fruit flies are a prime example of the many insects that live as larvae in plant material inhabited by both mutualistic and antagonistic microfungi. These flies, such as *Drosophila melanogaster*, transmit unicellular yeast fungi during oviposition to larval feeding sites (fruits) [9,10], which proliferate on the decaying plant tissue and serve as an essential dietary resource for the developing larvae [11,12]. Because most yeasts do not have active spore dispersal mechanisms and are particularly underrepresented in collections of airborne cells, insect vectors play an important role in the dispersal of such fungi, which may have favoured *Drosophila* -yeast mutualisms [9]. Filamentous fungi or moulds constitute another common type of microfungi in the *D. melanogaster* breeding habitat. *Drosophila* flies transmit *Aspergillus* , *Botrytis* , and *Penicillium* mould [13–15]; however, moulds are able to reach new habitat patches by means of wind-dispersed conidiospores. In contrast to yeasts, moulds appear to have a generally negative impact on *Drosophila* larval development. This impact is driven by insect density, priority effects, fungal species, and fungal toxins [16–18]. When their larval habitat is invaded by mould, *D. melanogaster* larvae suffer a sharp drop in fitness. This drop in fitness results from increased pre-adult mortality, or premature adult mortality as well as serious impairment of reproductive capabilities [19]. When larvae forage at high densities and mould colonies are at an early developmental stage, it is striking that the larvae are able to suppress mould growth [16,17]. Thus, the apparent attraction of larvae to mould colonies (Figure 1) may represent a defence against the noxious mould [20]. However, despite considerable impairment of fungal colony expansion by larvae, *Aspergillus* moulds appear to recover rapidly from the initially strong impact of insect attack [8].

In the context of inducible resistance, we address two novel suggestions for the attraction of *D. melanogaster* larvae to moulds: (i) Larval aggregation to mould colonies is an adaptive feeding response of a facultative fungivorous insect the aim of which is to extract essential nutrients from fungal tissue for sustaining larval development on an otherwise nutrient-poor fruit substrate. However, the ability of moulds to produce efficient insecticidal secondary metabolites confers strong resistance to fungivores and this harms the larvae (ii). Because the larvae feed initially on young hyphae but the final consequences are detrimental to insect fitness implies that the mould is able to shift to a more resistant phenotype that causes the serious negative effects on mould-confronted insects.

**Figure 2:**
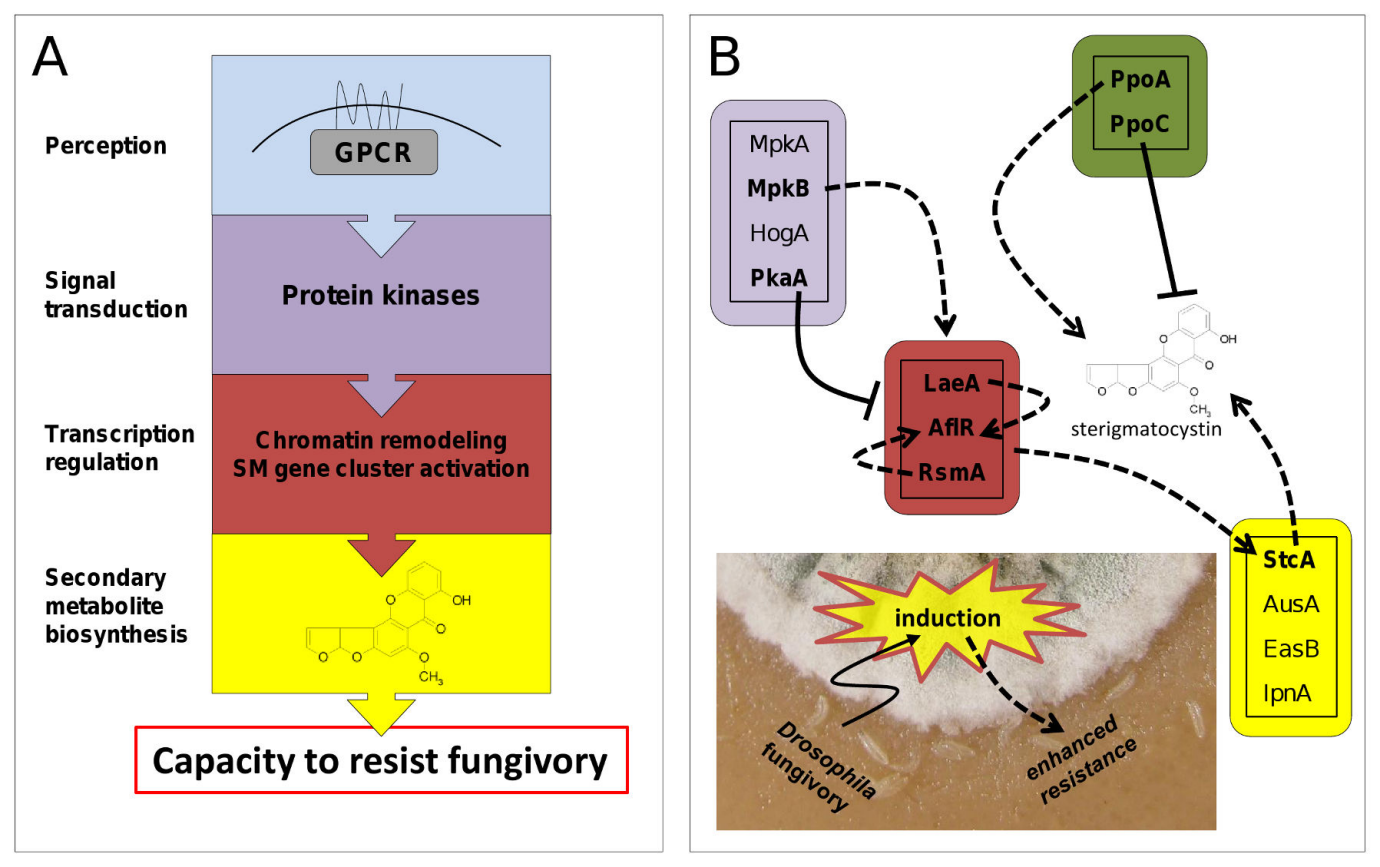
Pathways and molecular interactions potentially involved in *Aspergillus nidulans* induced defence response against *Drosophila melanogaster* larval grazing. (A) Molecular “modules” involved in defining the *A*. *nidulans* phenotype and hence the capacity of the mould to resist fungivory. Fungivore-specific signals may be perceived by G-protein coupled receptors (GPCR) that activate signal transduction pathways as determined by mitogen-activated protein kinase (MAPK) and protein kinase A (PKA) pathways. Signal transduction pathways interact with gene transcription regulators that operate on the level of epigenetic modifications (e.g. chromatin remodelling) and pathway-specific transcription factors controlling the coordinated activation of secondary metabolite (SM) gene clusters. (B) Molecular interaction network involved in triggering or suppressing properties of the chemical phenotype of *A*. *nidulans*. The green box indicates gene products involved in the generation of oxylipins (oxygenated products formed from polyunsaturated fatty acids), which regulate, through autocrine and paracrine signalling, fungal development and mycotoxin production. The white box depicts gene products of the biosynthesis pathways of some representative *A*. *nidulans* secondary metabolites. Given the positive influence of MpkA, LaeA, AflR, RsmA, StcA, and PpoA on the formation of insecticidal sterigmatocystin, the underlying genes (Table 1) were predicted to be up-regulated by *D*. *melanogaster* fungivory (dashed lines). Because PkaA and PpoC are thought to negatively affect mycotoxin formation, repression of the corresponding genes was expected (solid lines). Consult recent reviews by Bayram and Braus [21], Tsitsigiannis and Keller [22], and Brakhage [23] for detailed information on fungal secondary metabolite regulation and gene functions.

The secondary metabolism of filamentous fungi, that we propose underlies their resistance to fungivores, is tightly regulated (Figure 2) [21–23]. A key regulatory function of resistance to fungivory can be attributed to LaeA, a putative methyl transferase. It is involved in the global epigenetic control of many secondary metabolites in *Aspergillus* sp. and in other filamentous fungi [24–26]. LaeA is part of the VelB/VeA/LaeA protein complex (*velvet* complex) which synchronizes the biosynthesis of secondary metabolites with the development of *A. nidulans* [27]. Lots of *D. melanogaster* larvae die when exposed to wild type *A. nidulans* strains, but very few when larvae interact with LaeA or VeA loss-of-function *A. nidulans* mutants on a nutrient-rich substrate [8,28]. The *velvet* complex and hence its effect on the fungal phenotype is activated by a mitogen-activated protein kinase (MAPK) module that directly interacts with VeA in the nucleus [29]. Fungal MAPKs receive signals from G-protein coupled receptors that are able to perceive changes in environmental conditions. Ligands of cell surface receptors may include oxylipins, oxygenated polyunsaturated fatty acids, produced by the activity of dioxygenase enzymes that are encoded in *ppo* genes [30]. We suggest that the different molecular “modules” defining the *A. nidulans* phenotype (Figure 2A), and thus its capacity to resist *D. melanogaster* fungivory, are induced by insect feeding through changes in fungal gene expression (Figure 2B, Table 1). In analogy to herbivore-plant interactions [31], we consider changes in the fungal transcriptome a central process involved in mounting an induced defence that increases resistance to fungivores.

**Table 1:** Genes potentially involved in the inducible resistance of *Aspergillus nidulans* against *Drosophila melanogaster* larvae.

**Gene**	**Protein - Function**	**Biological process**	**Hypothesised expression changes**
**Signal transduction**			
*mpkA*	MAP kinase	Cell wall integrity signalling, polarized growth	?
*mpkB*	MAP kinase	Coordination of development and secondary metabolism	up-regulation
*hogA*	MAP kinase	Osmotic stress response, sexual development and sporulation	?
*pkaA*	Protein kinase	Conidiation, secondary metabolite regulation	down-regulation
**Regulation of gene transcription**			
*laeA*	Methyltransferase-domain protein	Chromatin re-modelling, control of development and secondary metabolism	up-regulation
*aflR*	C6 transcriptional activator	Regulation of sterigmatocystin pathway gene expression (e.g. *stcA*)	up-regulation
*rsmA*	bZIP transcription factor, binds to *aflR* promotor region	Regulation of sterigmatocystin pathway gene expression	up-regulation
**Oxylipin signalling**			
*ppoA*	Fatty acid dioxygenase	Oxylipin synthesis, sexual development, secondary metabolite regulation	up-regulation
*ppoC*	Fatty acid oxygenase	Oxylipin synthesis, asexual development, secondary metabolite regulation	down-regulation
**Secondary metabolite biosynthetic genes**			
*stcA*	Polyketide synthase	Sterigmatocystin biosynthesis	up-regulation
*ausA*	Polyketide synthase	Austinol and dehydroaustinol biosynthesis	?
*easB*	Polyketide synthase	Emericellamides biosynthesis	?
*ipnA*	Isopenicillin-N Synthase	Penicillin biosynthesis	?

We found that insect grazing on *A. nidulans* increased the resistance of this mould to subsequent fungivory. Changes in resistance to fungivorous insects were not only accompanied by differential expression of the secondary metabolite master regulator gene *laeA*, but also by the expression of several other genes involved in signalling, in oxylipin production and in the biosynthesis of various secondary metabolites. Our study thus provides first experimental evidence of a connection between changes in fungal resistance to insect grazing and candidate gene expression. These results demonstrate that fungi mount inducible defences.

## Results

### Insect-induced resistance to fungivory

**Figure 3:**
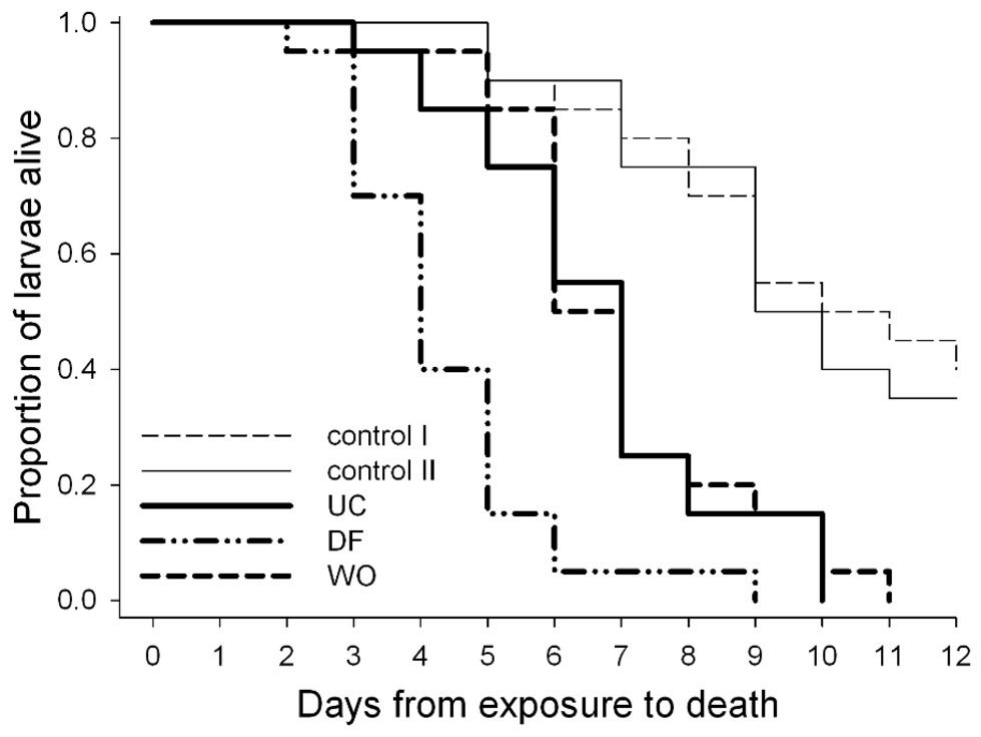
Insect grazing enhances the ability of *Aspergillus nidulans* to kill *Drosophila melanogaster* larvae. Daily survival of larvae exposed to mould colonies that were previously treated with conspecific larvae (DF), artificially wounded (WO), or remained untouched (UC). Fungal free fruit substrate without (control I) and with preceding presence of larvae (control II) were included to assess background mortality rates on the nutrient-poor fruit substrate.

To test whether *A. nidulans* colonies that had been fed on impair *D. melanogaster* larvae more efficiently than those that had not been fed on, single larvae were exposed to wild type colonies that had been previously confronted with (i) conspecific larvae, (ii) remained undisturbed or were (iii) artificially wounded. The effect of fungi on daily larval mortality was compared to mortality rates on fungal-free fruit substrate. Cox regression analyses revealed a grand effect of fungal infestation (χ^2^ = 14.02, P = 0.0002) and a significant interaction between fungal infestation and whether or not the mould had been challenged (χ^2^ = 4.09, P = 0.0432) (Figure 3). Compared to mould-free conditions, substrate infestation with *A. nidulans* significantly increased larval mortality rates (Wald χ^2^ = 29.92, P < 0.0001, hazard ratio: 4.02). In addition, the preceding presence of conspecifics further increased the chance of dying (Wald χ^2^ = 16.72, P = 0.0005, hazard ratio: 3.25). However, artificial wounding of mould colonies did not affect mortality rates (Wald χ^2^ = 0.10, P = 0.7556). Mortality rates were also unaffected by earlier feeding by conspecifics in mould-free fruit agar (Wald χ^2^ = 0.1732, P = 0.6773). *A. nidulans* is thus phenotypically plastic in its ability to resist, i.e. kill, *D. melanogaster* larvae.

### *A. nidulans* gene expression response to *D. melanogaster* larval grazing

**Figure 4:**
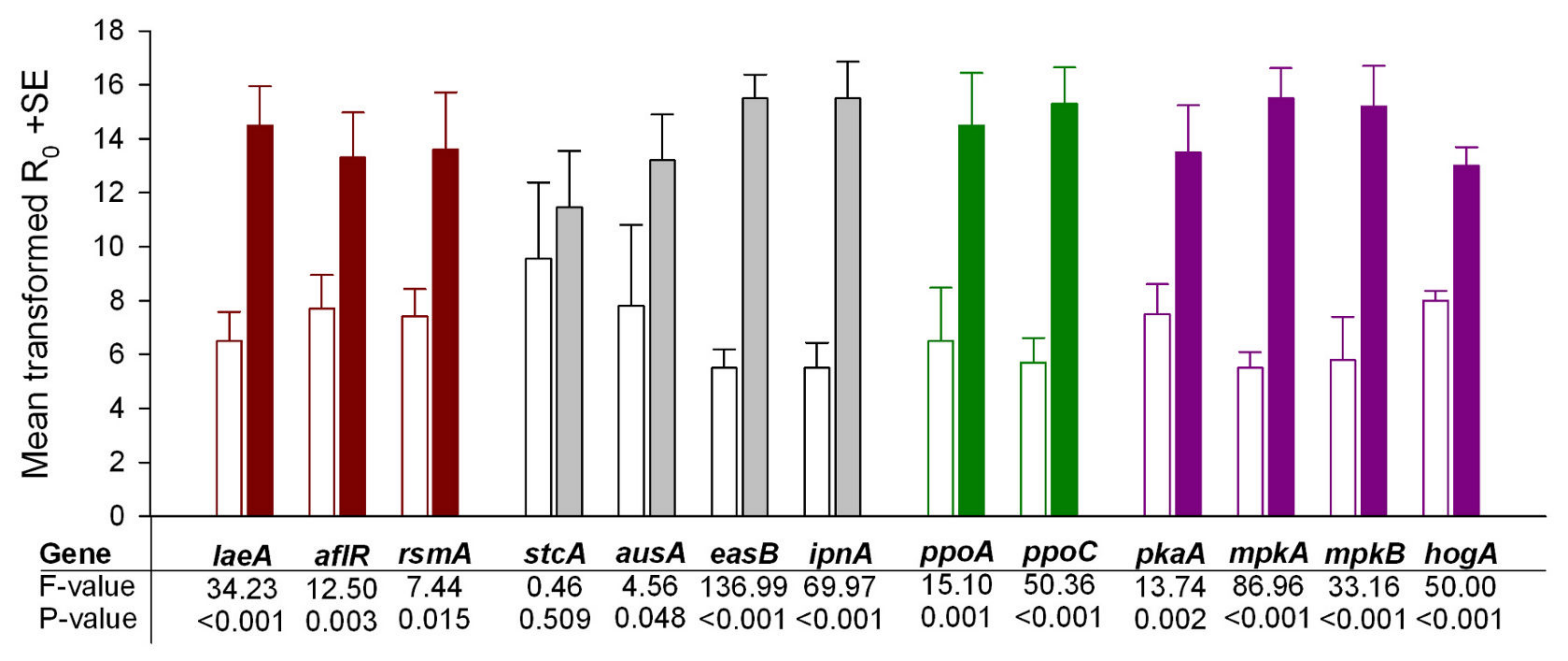
Influence of *Drosophila melanogaster* larval grazing on *Aspergillus nidulans* gene expression. Gene expression differences between wild type *A*. *nidulans* exposed to *D*. *melanogaster* larval grazing (filled bars) and unchallenged (open bars) colonies. Mean values are shown for initial SYBR green fluorescence amount (R_0_) proportional to initial amount candidate gene mRNA in the qRT-PCR runs (N = 5 per treatment). Colours represent different molecular “modules” involved in determining the fungal phenotype: transcription regulation (dark red), secondary metabolite biosynthesis (grey), oxylipin formation (green), signal transduction (violet) (see Figure 2 for details). Data were transformed to eliminate gene-specific variation in expression differences and to achieve normality and variance homogeneity; see Methods for details and Figure S2 for untransformed gene expression data. Statistics refer to results of the between-subject effect analysis of the multivariate nested analysis of variance on ranks; *Drosophila* larval grazing had an overall effect on fungal gene expression, P = 0.025.

We quantified mRNA levels of several genes to test if induced resistance to insect grazing is accompanied by differential expression of genes known to be involved in determining the (chemical) phenotype of *A. nidulans* (Figure 2, Table 1). The expression of genes representative of the different “modules” putatively involved in resistance to fungivores was indeed affected by *D. melanogaster* larval grazing (Figure 4). These genes have functions in secondary metabolite gene expression regulation, secondary metabolite biosynthesis, oxylipin formation, and signal transduction. Twelve out of the thirteen genes tested were significantly up-regulated. Only one biosynthetic gene was not differentially expressed (Figure 4). This gene encodes an early step of the sterigmatocystin pathway (*stcA*). Even though expression of some of the candidate genes might be expected to be repressed by fungivory (e.g. *ppoC* or *pkaA*, see Figure 2 and Table 1), they exhibited significantly enhanced expression when *A. nidulans* was exposed to grazing. As to the *a priori* knowledge of the gene’s functional impact the data indicate the induction of a substantial fungal phenotypic shift in response to insect grazing.

### *D. melanogaster* larval feeding on wild type and chemical deficient (ΔlaeA) *A. nidulans*

**Figure 5:**
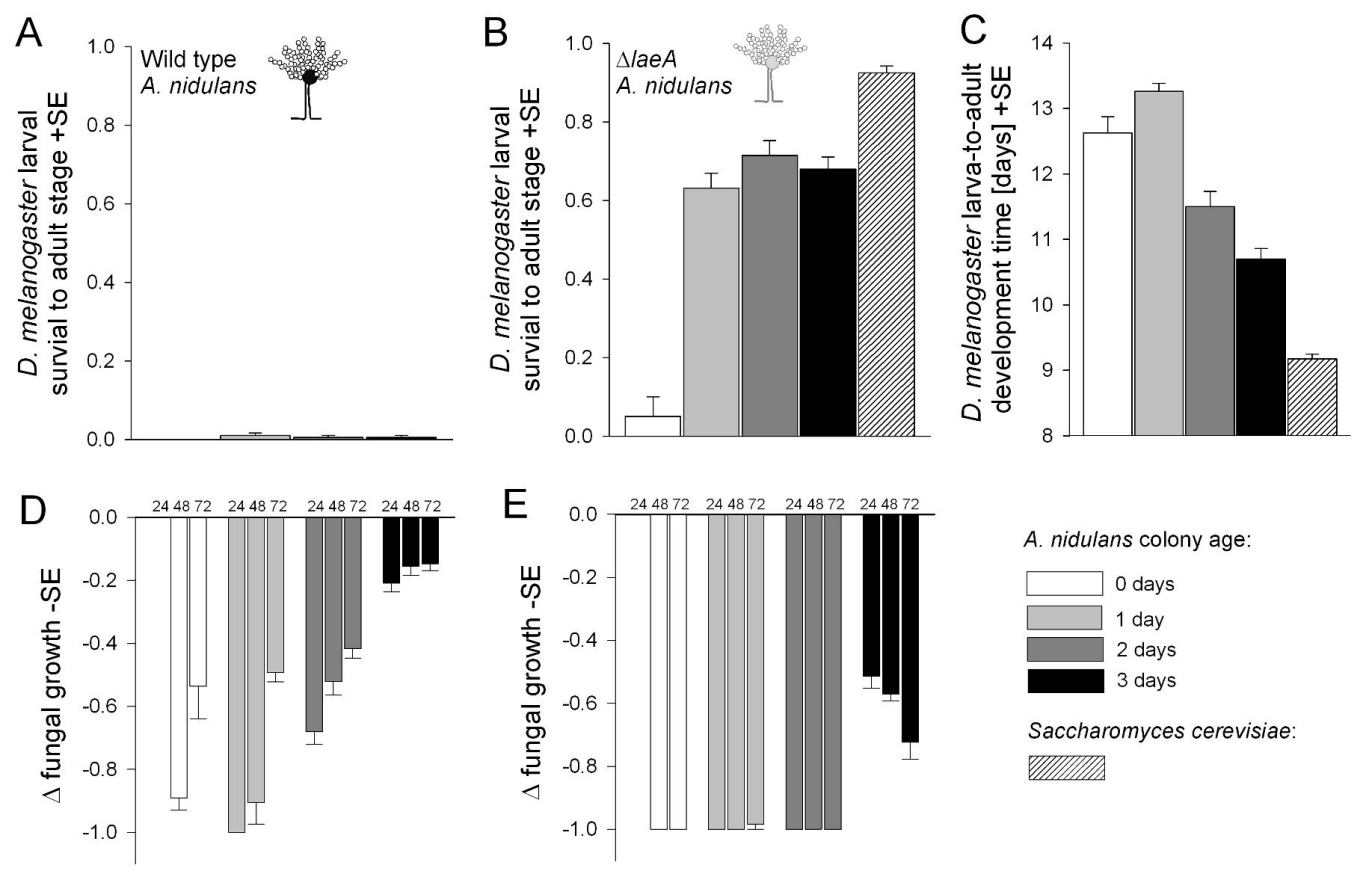
Reciprocal fitness consequences of *Drosophila melanogaster* larval feeding on *Aspergillus nidulans.* (A) and (B) Mean survival of *D*. *melanogaster* larvae to the adult stage on wild type, chemical deficient Δ*laeA A*. *nidulans*, and yeast *Saccharomyces cerevisiae*. Availability of potential *A*. *nidulans* food to larvae was controlled by varying the time between inoculation of conidia and transfer of larvae (A. *nidulans* age: 0 to 3 days). Initial yeast inoculum was 1000 cells. (C) Mean larva-to-adult development times on Δ*laeA* and *S*. *cerevisiae*. (D) and (E) Mean suppression of wild type and Δ*laeA A*. *nidulans* surface growth relative to undisturbed control colonies 24, 48 and 72 hours after the transfer of larvae. Δ-values may range from 0 (no suppressive influence of insect grazing relative to undisturbed colonies) to -1 (100% suppression of mould development). See text for statistical details.

To test whether the LaeA-dependent ability of resisting fungivore attack is the key fungal trait that deters *D. melanogaster* larvae from using *A. nidulans* as food, we offered larvae a chemical deficient Δ*laeA* or a wild type *A. nidulans* strain as the only microfungal diet on sterile fruit substrate and recorded larval development and fungal growth. Wild type *A. nidulans* strain scarcely supported development to the adult (Figure 5A) and fungal-free control substrate did not support development at all (data not shown). In contrast, larvae developed into adult flies on the chemical deficient Δ*laeA* mutant strain, yet survival was not as high as on fruit agar infested with *S. cerevisiae* (Figure 5B). Survival was very low in the treatment where *A. nidulans* had no developmental “head start”, and initial fungal biomass was virtually zero (generalized linear model, P < 0.0001). Even though survival probabilities were the same in the other Δ*laeA A. nidulans* treatments, development time to the adult stage was negatively related to fungal age and hence the amount of fungal food available to the larvae (P < 0.0001; Figure 5C). Compared with development on three-day old *A. nidulans*, time to adult eclosion was significantly shorter when larvae were offered *S. cerevisiae* (P < 0.0001).

Larvae strongly suppressed the development of Δ*laeA*, and the fungus did not recover, i.e. there was no positive relationship between changes in fungal growth and time (24, 48, 72 hours) after larval transfer. Although hyphal fragments were visible, the development of aerial hyphae was almost completely eliminated in zero to two-day old colonies (Figure 5E). Aerial hyphae were clearly visible in three-day old colonies but suppression tended to increase over time (repeated mixed model, P = 0.0025; Figure 5E). Moreover, we observed no conidiophore (asexual reproductive organs) formation in insect-challenged colonies. The wild type strain, however, was less strongly affected and clearly recovered from larval feeding activity with increasing time (P < 0.0001; Figure 5D). All replicate colonies also developed conidiospores.

In contrast to the generally negative influence of insects on mould growth, the dietary yeast *S. cerevisiae* reached higher cell population densities when exposed to *D. melanogaster* larvae feeding compared to a non-insect treatment (Figure S3). Thus, these results reveal two important aspects of *Drosophila* fungivory: (i) filamentous mould, if impaired in secondary metabolite production, can serve as a suitable but rapidly diminishing resource, and fungivory entails costs of strong insect competition, (ii) unicellular yeast is a re-growing food source that is effective in mediating high insect fitness.

## Discussion

In the present study, we show that *A. nidulans* kills *D. melanogaster* larvae more rapidly if the mould had been previously exposed to insect grazing. The enhanced anti-fungivore properties were not observed in artificially wounded colonies. This suggests the existence of insect-specific elicitors triggering the induction of greater resistance. This inducible resistance to fungivory reveals the previously unknown flexibility of a saprotrophic fungus in its ability to harm a fungivore.

Here we show that the development of induced resistance to insect grazing coincides with a fundamental shift in fungal gene expression. In line with previous assumptions that the epigenetic regulator of secondary metabolite formation, LaeA, plays a central role in controlling resistance to fungivory [8,32] the corresponding gene, *laeA*, was significantly up-regulated in *D. melanogaster*-challenged colonies. Genes encoding regulatory elements of the sterigmatocystin gene cluster, *aflR* and *rsmA*, were also up-regulated. Overexpression of *rsmA* has recently been shown to result in enhanced sterigmatocystin biosynthesis coupled with strong feeding avoidance by fungivorous collembolans [33], and sterigmatocystin strongly affects *D. melanogaster* development [34]. Despite the apparent activation of regulatory elements of the sterigmatocystin cluster it is interesting that *stcA* which encodes an early biosynthetic enzyme of the sterigmatocystin pathway was not differentially expressed. This indicates that the sterigmatocystin gene cluster was not activated at this early stage of interaction with the fungivore. Deactivation of the sterigmatocystin pathway does not reduce resistance to insect grazing [28]. Instead, sterigmatocystin deficient mutant moulds caused higher *D. melanogaster* larval mortality than the wild type. This difference strongly suggests the involvement of other secondary metabolites in *A. nidulans* resistance to fungivorous insects. In line with this assumption, we found in the present study that genes encoding enzymes of other secondary metabolite pathways were more highly expressed in the *Drosophila* treatment. The gene *easB* is involved in the production of emericellamides and *ipnA* in that of penicillin. Penicillin and some emericellamides have antimicrobial properties [35,36]. The polyketide synthase gene, *ausA*, is involved in the formation of meroterpenoids, austinol and dehydroaustinol [37]. Related compounds isolated from *Penicillium* *brasilianum* appear to have insecticidal properties [38]. Given that *A. nidulans* harbours many more verified and putative secondary metabolite pathways [39] more biosynthetic genes and their regulatory elements might be activated by *D. melanogaster* fungivory. Decoding the cryptic diversity of secondary metabolites induced by insects will be a major future task in establishing a chemical compound-based principle of defence against fungivores.

Independently of their demonstrated effects on secondary metabolite production (Figure 2, Table 1), the expression of genes involved in MAPK and PKA signalling was enhanced in *D. melanogaster*-challenged *A. nidulans* colonies. Although protein kinase activity measurements are required for disentangling the relative contribution of each signalling cascade to the induced phenotype, our data suggest that insect fungivory triggers a reshuffle of large parts of the signalling network. We postulate that accumulation of signal amplifiers is a prerequisite for enhanced defence gene expression and development of induced resistance to fungivores [40].

Enhanced expression of *ppoA* and *ppoC* in *D. melanogaster*-challenged colonies suggests that changes in the oxylipin profile have a role in mediating resistance to fungivory. Overexpression of *ppoA* amplifies mycotoxin production and is accompanied by higher *D. melanogaster* larval mortality, relative to wild type *A. nidulans* [28]. The *ppoC*-derived fatty acid oxygenase contributes to the production of volatile compounds, such as 1-octen-3-ol [41], that have been shown to have neurotoxic effects on *D. melanogaster* [42]. Thus, in addition to mycotoxin regulation via the hormone-like effects of oxylipins, a direct impact on fly larvae may affect *D. melanogaster* development. Changes in oxylipin production may also be part of an injury-response mechanism [43] triggered by grazing larvae, which may have an additional regulatory influence on the *A. nidulans* phenotypic shift. Note, however, that a mechanical challenge alone was not sufficient to enhance the capacity of the mould to resist fungivory (Figure 3). Likewise, artificial wounding does not induce the putatively anti-fungivore lectin proteins of *Coprinopsis* *cinerea* although fungivorous nematodes do [44].

Deficiencies in the *A. nidulans* velvet complex, Δ*laeA* or Δ*veA*, significantly reduce *D. melanogaster* mortality on nutrient-rich medium [8,28], and attraction of larvae was interpreted as an anti-fungal defence [20]. Our fly larvae were able to exploit *A. nidulans* as food on an otherwise nutrient-poor fruit substrate when the fungus is unable to respond to feeding by expressing *laeA*. Compared to *S. cerevisiae*, however, the chemical deficient *A. nidulans* is a rapidly diminishing fungal food that causes strong larval competition. The amount of food declines because the mould colony does not persist. The failure to persist is probably because persistence and proliferation requires the maintenance of tissue integrity. Tissue integrity is completely disrupted, however, by the *D. melanogaster* larvae and their movement through the medium prevents the re-growth of the fragmented fungal tissue. The dramatic consequences of fungivory on undefended mould (as in our chemically deficient strain) would have favoured the evolution of efficient, secondary metabolite-based, resistance to insect grazing. Strong chemical defences have been proposed as being typical for many filamentous microfungi exploiting short-lived resources on which they face interactions with multiple antagonists [45].

## Conclusion

Our experiments provide novel evidence of an adaptive connection between induced resistance against fungivore grazing and the expression of fungal signal transduction, epigenetic regulation and secondary metabolite biosynthesis pathways. *Aspergillus nidulans* perceives grazing by insects and adjust its phenotype to maintain high fitness in fungivore-rich habitats. In contrast to the growth stimulating effect of insect feeding activity on a yeast mutualist, effective eradication and ingestion of chemical deficient Δ*laeA A. nidulans* by insect larvae suggests that induced resistance in the mould may have evolved in response to an antagonistic arms race with fungivorous arthropods.

## Materials and Methods

### Culture of organisms and general experimental conditions

We used a *D. melanogaster* culture that originated from 113 isofemale lines caught in Kiel, Germany, in 2006. They were cultured and sterile larvae were prepared following our standard methods [8]. Except for the fungal gene expression experiment, all insect-fungus confrontation experiments were conducted using a banana-agar medium (50% mashed banana/50% demineralised water, v/v) [46]. Our *A. nidulans* strains were a wild type (RDIT 2.3) and a chemical deficient Δ*laeA* mutant (RJW 46.4) in same veA1 genetic background [24]. Cultivation of fungi and conidia harvesting procedures followed published protocols [8]. All the experiments were incubated at 25°C and constant darkness.

### Insect-induced resistance to fungivory

To test whether *D. melanogaster* larvae mediate induced resistance in *A. nidulans*, we compared the survival of individual larvae on untouched control colonies and colonies previously challenged with conspecific larvae. For this test, 2 ml microtubes filled with banana agar were inoculated with wild type *A. nidulans* conidia [8,46]. After 24 hours incubation a single larva was added to each tube. These larvae were removed after a further 24 hours and replaced by a new one. At the same time, larvae were added to unchallenged colonies. In addition, we recorded the survival of individual larvae in fungal free substrate under two treatments. One treatment was without previous feeding by conspecific larvae and the other with previous feeding. To test whether physical damage alone is sufficient to induce resistance in *A. nidulans* independently of larval foraging, we included a wounding treatment. For this, 36 hours after inoculation with conidia, the hyphal mats were touched four times with autoclaved needles to produce marks resembling the “chew marks” left by larvae (see Figure S1). Twelve hours later larvae were added. We then recorded whether larvae were alive or dead every day for a maximum of 14 days after inoculation.

### *D. melanogaster* larval feeding on wild type and chemical deficient (ΔlaeA) *A. nidulans*

Experimental units containing wild type or chemical deficient Δ*laeA A. nidulans* of different age were prepared [8,46]. Fungal age at larval transfer was manipulated in order to provide different amounts of fungal tissue for the larvae. Ten first-instar larvae were added to each experimental unit and the unit then sealed with a sterile cotton plug. In parallel, a mould-free control treatment and a treatment with yeast, *Saccharomyces cerevisiae* [46], were set up. There were N = 200 replicates in total with N = 20 replicates for each treatment. We counted the number of emerging flies and recorded the time (days after larval transfer) when the flies eclosed from their puparia. Following the established protocol [8], we quantified mould growth 24, 48 and 72 hours after the introduction of the larvae by image analysis.

### *A. nidulans* gene expression response to *D. melanogaster* larval grazing

To obtain fungal tissue from insect-challenged and unchallenged treatments, *A. nidulans* was inoculated on KOH-treated, sterile cellophane sheets placed on malt extract agar plates (35 mm in diameter, filled with 3 ml medium). 100 µl conidia suspension (10,000 conidia/µl) were added and each plate was rotated to spread the suspension evenly across the cellophane. Plates were closed with the lids and incubated at 25°C for 24 hours. Subsequently, 40 sterile *D. melanogaster* larvae were released onto each plate and incubation continued for another 24 hours. After this period, larval damage could be clearly seen as elongated “chew marks” (see Figure S1). Fungal tissue was removed with a scalpel from unchallenged treatments and those challenged by *D. melanogaster* larval grazing. Fungal tissue from three colonies was pooled to generate one biological replicate (in total five biological replicates per treatment). The tissue was shock-frozen in liquid nitrogen and lyophilized for approximately 24 hours. Lyophilized and powdered tissue (10 mg) was treated with 1 ml TRIzol® Reagent (Ambion) for RNA extraction, according to the manufacture’s instructions. The remaining DNA was digested using TURBO DNA-*free* (Ambion). RNA quantity and quality were determined by measuring absorbance at A260/A280 and formaldehyde de-naturating agarose (0.65%) gel. The efficiency of DNA digestion was checked by standard PCR combined with polyacrylamid gel analysis.

### Quantitative RT-PCR

Forward (F) and reverse (R) oligonucleotide primer sequences used in qRT-PCR experiments and amplicon properties are listed in Table S1. Sequence information was retrieved from exon only sequences provided by http://www.aspgd.org/. We designed primers using Primer Premier (Premier Biosoft). Primers were synthesized and provided by Eurofins (http://www.eurofins.de). Primer suitability was tested on *A. nidulans* reference DNA, and amplicon identity was verified by complete melting curve analysis and amplicon sequencing. We used an external luciferase control RNA spike (Promega, L4561) for normalizing candidate RNA quantification rather than common fungal house-keeping genes, as suggested by Rehrig et al. [47]. This was done to avoid the likelihood that the expression of house-keeping genes is affected by ecological challenges such as insect grazing [47]. This was particularly relevant for our experiments because insect fungivory has not yet been used as an ecological factor affecting fungal gene expression. No homologues were revealed by blasting the luciferase primer sequences against the *A. nidulans* genome. We used One-Step qRT-PCR (Qiagen) according to the manufacture’s instruction to reverse transcribe the candidate RNA and amplified the cDNA on a Strategen Mx3000P engine (Agilent) using SYBR Green (Lonza) under the PCR conditions: 50°C for 30 min, 95°C for 15 min, followed by 35 cycles of 95°C denaturation for 15 s and 56°C annealing for 30 s and 72°C extension for 30 s. All qRT-PCR runs were followed by a melting curve analysis to verify proper amplification of the candidate genes. Each sample was run in duplicate (N = 260 amplifications in total). We determined PCR efficiencies (E), threshold fluorescence (R_CT_-values), and initial fluorescence (R_0_) directly from the PCR kinetic curves using LinRegPCR 12.12 (http://LinRegPCR.HFRC.nl) [48]. To statistically compare (see below) the expression of candidate genes in unchallenged and *Drosophila* fungivory samples, we used R_0_, equivalent to the initial amount of candidate mRNA in a sample [49], as a quantitative measure of the strength of gene expression under the particular experimental conditions. For the results of each qRT-PCR the quantity equivalent, R_0_, of the candidate gene was considered relative to independent R_0_ for the reference gene. The resulting R_norm_(unchallenged) is the initial fluorescence of the candidate gene normalized to the reference gene in the unchallenged sample and R_norm_(insect grazing) that in the insect challenged sample.

### Statistical analyses

Larval developmental success, fungal growth, and daily larval survival were analysed using generalized linear models in SAS 9.3 [8]. Two factors are likely to affect overall variation in the expression of candidate *A. nidulans* genes. These are fungivory by *D. melanogaster* larvae and constitutive gene-specific differences. In order to test the effect of larval fungivory separately from that of constitutive gene-specific differences, we removed gene-specific effects by standardizing individual R_0_ values. To do this, the mean R_0_ for all replicates of candidate gene *x* was subtracted from the individual R_0_ of each candidate gene *x*. The resulting values were subsequently divided by the standard deviation for all replicates of candidate gene *x* [50]. Because normality and variance homogeneity criteria were not met, we assigned standardized R_0_ values to ranks to achieve both normality and homogeneous variances. We applied a multivariate general linear model to test the influence of *D. melanogaster* feeding on *A. nidulans* gene expression. The factor “insect treatment” was nested within “technical replicate” to account for variation between technically replicated qRT-PCR runs.

## Supporting Information

Figure S1 **Effect of 20 h *D. melanogaster* larval grazing on the appearance of 48 h old *A. nidulans* (B), compared to undisturbed fungal growth (A).** Bright areas indicate the presence of hyphal mats. In both the unchallenged control and the *Drosophila* larval grazing treatment, fungi developed conidiophores and were at the initial stage of conidiospore production; yet greenish pigments were not visible to the naked eye. Arrows point at *D. melanogaster* larvae that left “chew marks” (dark areas where the cellophane/culture medium shines through) on the fungus. Images were taken with dark field adjustment using a stereomicroscope (Discovery V8, Zeiss, Germany) equipped with a digital camera system (AxioCam Icc1, Zeiss, Germany).
(TIF)

Figure S2 **Untransformed mean R_0_ values proportional to *Aspergillus nidulans*** **candidate gene expression differences**. Normalised R_0_ values are representative of the initial candidate mRNA levels in the treatment samples.
(TIF)

Figure S3 ***Saccharomyces cerevisiae* cell population development with and without the influence of *Drosophila melanogaster* larval feeding.** To quantify the influence of *D. melanogaster* fungivory on growth of *S. cerevisiae* 2 ml microtubes were filled with 1 ml banana agar. They were then inoculated with 10,000 cells of *S. cerevisiae* (strain DSM 70449 obtained from the DSMZ, German Collection of Microorganisms and Cell Cultures, Braunschweig, Germany) in 1 µl NaCl solution. Directly after inoculation, ten *D. melanogaster* larvae were added to each tube. From three cohorts (after 24, 36 and 48 hours incubation) of N = 7 randomly chosen replicates for each treatment yeast cells were washed off the substrates surface. For this, the same 1 ml saline solution was repeatedly (15 times) pipetted into each tube to thoroughly flush out the yeast cells. To remove large particles and larvae the cell suspension was filtered through a double layer of Miracloth^®^ and yeast cell population sizes were analysed by means of flow cytometry (BD AccuriC6 Flow Cytometer, BD Biosciences, U.S.A.). We specified a fast flow rate and set the run limits to 10,000 events. Compared to larval-free yeast control washes we specified the polygonal area for particle quantification. Generalized linear model; larvae, time, larvae*time interaction, all P < 0.0001; post hoc comparison at time 48 h, larvae: P < 0.0001.
(TIF)

Table S1 **Primer list.**
(DOCX)
