# Supplementary figures and images for: Induced Fungal Resistance to Insect Grazing: Reciprocal Fitness Consequences and Fungal Gene Expression in the Drosophila-Aspergillus Model System
Source: PLoS One. 2013 Aug 30;8(8):e74951. doi: 10.1371/journal.pone.0074951 (PMC3758311; doi:10.1371/journal.pone.0074951)

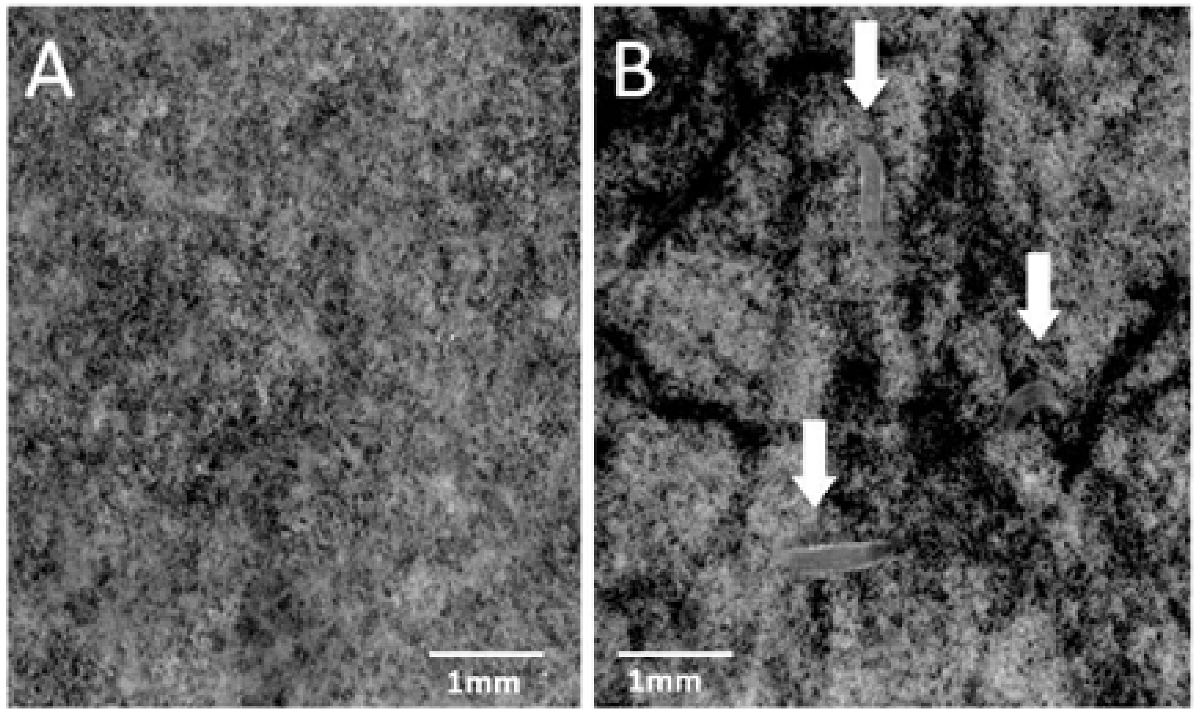

Supplement: Figure S1 — Effect of 20 h D. melanogaster larval grazing on the appearance of 48 h old A. nidulans (B), compared to undisturbed fungal growth (A). Bright areas indicate the presence of hyphal mats. In both the unchallenged control and the Drosophila larval grazing treatment, fungi developed conidiophores and were at the initial stage of conidiospore production; yet greenish pigments were not visible to the naked eye. Arrows point at D. melanogaster larvae that left “chew marks” (dark areas where the cellophane/culture medium shines through) on the fungus. Images were taken with dark field adjustment using a stereomicroscope (Discovery V8, Zeiss, Germany) equipped with a digital camera system (AxioCam Icc1, Zeiss, Germany). (TIF) [file pone.0074951.s001.tif]

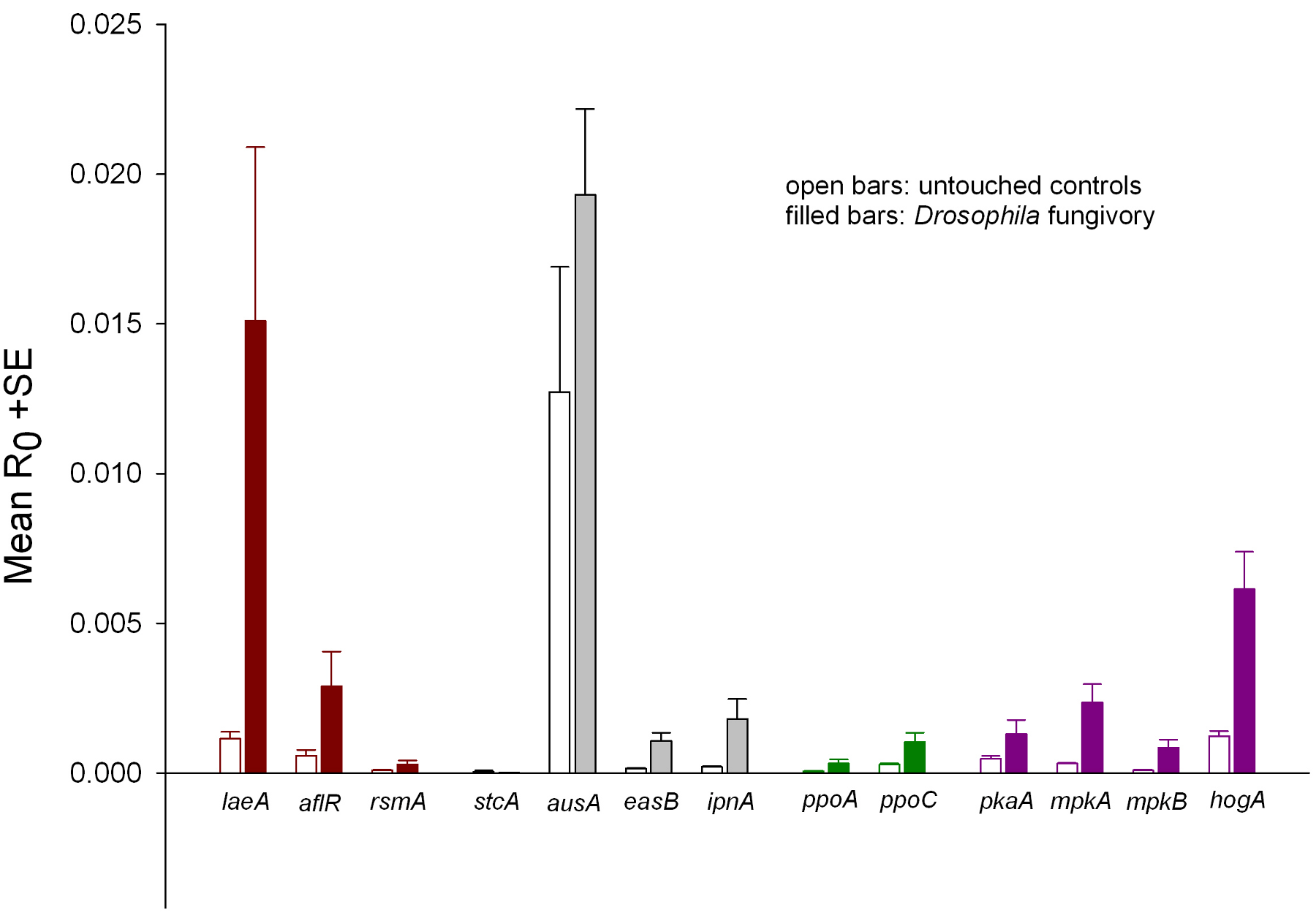

Supplement: Figure S2 — Untransformed mean R0 values proportional to Aspergillus nidulans candidate gene expression differences. Normalised R0 values are representative of the initial candidate mRNA levels in the treatment samples. (TIF) [file pone.0074951.s002.tif]

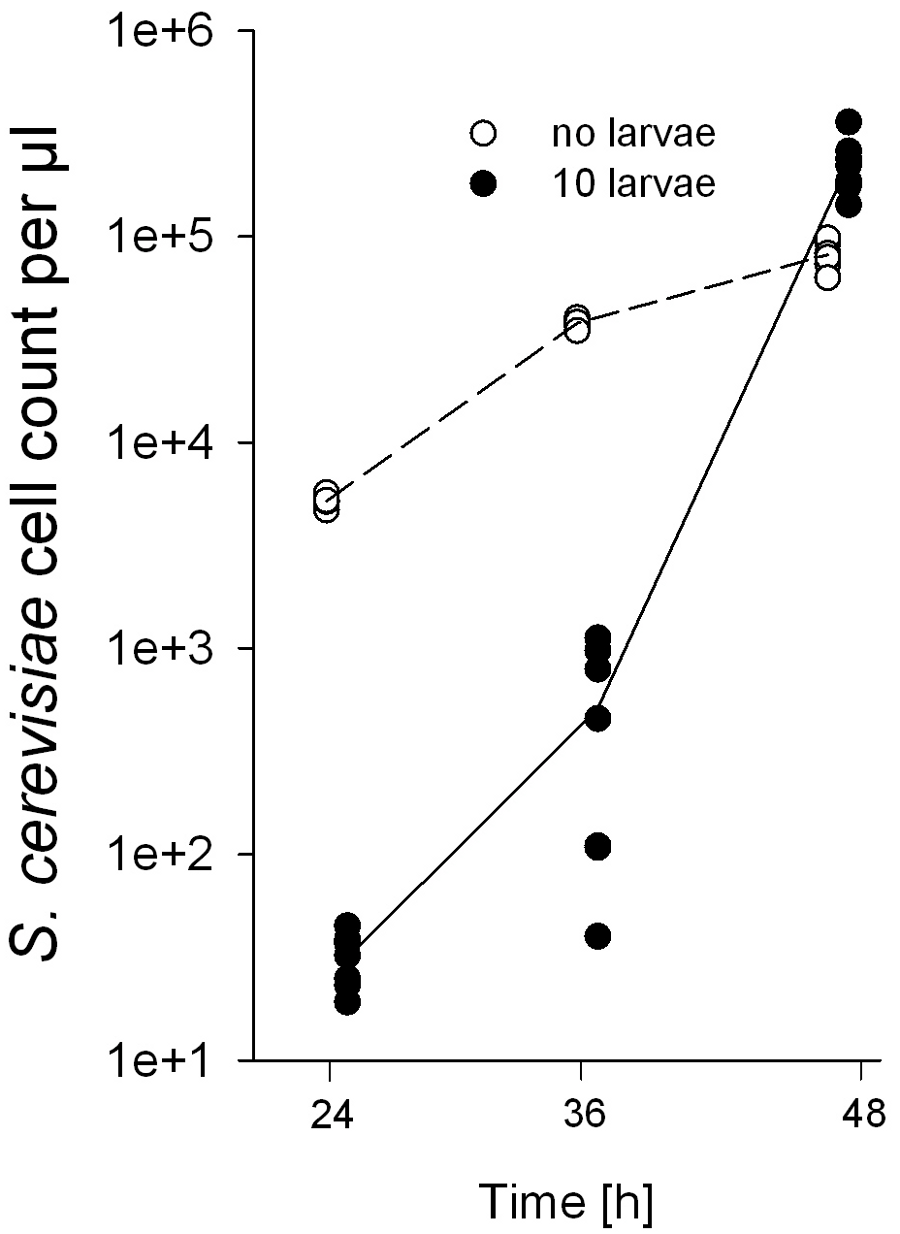

Supplement: Figure S3 — Saccharomyces cerevisiae cell population development with and without the influence of Drosophila melanogaster larval feeding. To quantify the influence of D. melanogaster fungivory on growth of S. cerevisiae 2 ml microtubes were filled with 1 ml banana agar. They were then inoculated with 10,000 cells of S. cerevisiae (strain DSM 70449 obtained from the DSMZ, German Collection of Microorganisms and Cell Cultures, Braunschweig, Germany) in 1 µl NaCl solution. Directly after inoculation, ten D. melanogaster larvae were added to each tube. From three cohorts (after 24, 36 and 48 hours incubation) of N = 7 randomly chosen replicates for each treatment yeast cells were washed off the substrates surface. For this, the same 1 ml saline solution was repeatedly (15 times) pipetted into each tube to thoroughly flush out the yeast cells. To remove large particles and larvae the cell suspension was filtered through a double layer of Miracloth® and yeast cell population sizes were analysed by means of flow cytometry (BD AccuriC6 Flow Cytometer, BD Biosciences, U.S.A.). We specified a fast flow rate and set the run limits to 10,000 events. Compared to larval-free yeast control washes we specified the polygonal area for particle quantification. Generalized linear model; larvae, time, larvae*time interaction, all P < 0.0001; post hoc comparison at time 48 h, larvae: P < 0.0001. (TIF) [file pone.0074951.s003.tif]
