# Supplementary material for: Induced Fungal Resistance to Insect Grazing: Reciprocal Fitness Consequences and Fungal Gene Expression in the Drosophila-Aspergillus Model System
Source: PLoS One. 2013 Aug 30;8(8):e74951. doi: 10.1371/journal.pone.0074951 (PMC3758311; doi:10.1371/journal.pone.0074951)
Supplement: Table S1 — Primer list. (DOCX) [file pone.0074951.s004.docx]

| **Table S1. Primer list.** | | | |
| --- | --- | --- | --- |
| **Gene**  **(ID)** | **‘5 →3’** | **Predicted amplicon size** | **Melting temperature T_m_ (°C)** |
| *aflR*  (AN7820) | GTCTCCGAATACTTCCACCT (F)  ATGCCATCCATACCCTCA (R) | 114 bp | 86.0 |
| *ausA*  (AN8383) | TGCTGTCCGCTACCTTC (F)  AGACCTGCTCTGCCTCC (R) | 102 bp | 86.8 |
| *easB* (AN2547) | AAAGCCAAGCCCGTTAG (F)  CGTAGTGCCTGCGAGAA (R) | 137 bp | 88.0 |
| *hogA* (AN1017) | TTGCCGAAGTTCCTGG (F)  TCATCTGTCTCATCGTGGTAT (R) | 176 bp | 88.3 |
| *ipnA* (AN2622) | CTTGCGATTCGTGCCTAC (F)  GCGTGAAGTTCGGGTT (R) | 118 bp | 87.7 |
| *laeA*  (AN0807) | GCTCCTATTCAGCCTCCG (F)  ATGACACTACCGCAACCC (R) | 131 bp | 89.6 |
| *mpkA*  (AN5666) | CACTTTGAGGTGGTTGACG (F)  TGTTGCCTATTTGCTGTTG (R) | 130 bp | 89.4 |
| *mpkB*  (AN3719) | CGGGAAGCCCTTGTT (F)  CCGTCGGGACTTGATT (R) | 109 bp | 85.0 |
| *pkaA* (AN6305) | AACCACCGCTACTATGCC (F)  GGAAAGGATGCCTGACG (R) | 115 bp | 84.7 |
| *ppoA* (AN1967) | GATTATGTCCGAACGATTT (F)  CGGTTGCCATTGCTG (R) | 112 bp | 83.4 |
| *ppoC* (AN5028) | TCAGTGCCAACGACGAG (F)  TGCCAAGACCCATAAGC (R) | 102 bp | 88.0 |
| *rsmA*  (AN4562) | CGACGAGTTTCTTGCTGTG (F)  CGGCGAGTAAGTTTGAGGATT (R) | 197 bp | 89.8 |
| *stcA* (AN7821) | AAATCGCCGCAGTTCATC (F)  GCAACAAGCCACTTATTCTCAA (R) | 155 bp | 88.0 |
